# Association of the sense of coherence with physical and psychosocial health in the rehabilitation of osteoarthritis of the hip and knee: a prospective cohort study

**DOI:** 10.1186/1471-2474-14-159

**Published:** 2013-05-04

**Authors:** Thomas Benz, Felix Angst, Susanne Lehmann, André Aeschlimann

**Affiliations:** 1 Research department, Rehabilitation clinic “RehaClinic”, Bad Zurzach, 5330, Switzerland

**Keywords:** Hip, Knee, Osteoarthritis, Sense of coherence, Rehabilitation, Physical and psychosocial health

## Abstract

**Background:**

According to Antonovsky’s salutogenic concept, a strong sense of coherence is associated with physical and psychological health. The goal of this study was to analyze the association of Antonovsky’s sense of coherence with physical and psychosocial health components in patients with hip and knee osteoarthritis before and after in- and outpatient rehabilitation.

**Methods:**

Prospective cohort study with 335 patients, 136 (41%) with hip and 199 (59%) with knee osteoarthritis. The outcome was measured by Short Form-36 (SF-36), Western Ontario and McMaster Universities Osteoarthritis Index (WOMAC) and the Sense of Coherence (SOC-13). Baseline scores of the SF-36 and WOMAC scales and the observed effect sizes after rehabilitation were correlated with the baseline SOC-13. These correlations of the SF-36 scales were compared to the Factor Score Coefficients for the Mental Component Summary of SF-36, which quantify the factor load on the psychosocial dimension. Predictive impact of the baseline SOC-13 for the SF-36 and WOMAC scales (baseline scores and effect sizes) was then determined by multivariate linear regression controlled for possible confounders.

**Results:**

At baseline, the SOC-13 correlated with the WOMAC scores between r = 0.18 (stiffness) and r = 0.25 (pain) and with the SF-36 scores between r = 0.10 (physical functioning) and r = 0.53 (mental health). The correlation of these SF-36 correlation coefficients to the Factor Score Coefficient of the SF-36 Mental Component Summary was r = 0.95. The correlations for the effect sizes (baseline → discharge) with the baseline SOC-13 global score were all negative and varied between r = 0.00 (physical functioning) and r = −0.19 (social functioning). In the multivariate linear regression model, the explained variance of the SF-36 scores by the baseline SOC-13 increased continuously from physical to psychosocial health dimensions (from 12.9% to 29.8%). This gradient was consistently observed for both the baseline scores and the effect sizes. The results of the WOMAC were consistent with the physical health scales of SF-36.

**Conclusions:**

The sense of coherence was associated with psychosocial health dimensions but hardly with physical health. The higher the load of a scale on the psychosocial dimension the higher was its correlation to the sense of coherence. This is in contrast to the idea of Antonovsky who predicted high associations with both mental and physical health.

## Background

Osteoarthritis (OA) is one of the most expensive and disabling musculoskeletal diseases in the developed countries [1]. The knee and hip are the large joints most prevalently affected by OA [2,3]. OA reduces mobility, influences quality of life, and the ability of work as well as psychosocial interaction [1,4]. In other words, OA has an impact on the biopsychosocial health and on the patients experience of the disease severity.

The management of OA of the hip and knee recommended by the European League against Rheumatism (EULAR) includes medication, exercise therapy, patient education and joint replacement [1,2]. Rehabilitation can improve physical function in the mid-term and pain in the long term [5]. Little is known about the factors influencing the outcome of rehabilitation based on the recommendations by EULAR (with exclusion of arthroplasty). Biomedical factors play an important role in the development and progression of the disease, whereas psychosocial factors may have greater implications in determining the overall level of disability of affected individuals [6]. Depressive symptoms have been identified as a negative predictor of response to rehabilitation [7] and are a risk factor for adverse long term pain-related outcomes such as physical disability, increased severity of pain, enhanced pain sensitivity [8]. Psychosocial variables influence functional improvements and effects of rehabilitation in patients with various diagnoses [9,10]. Nevertheless, psychosocial factors and the influence of salutogenic health factors are rarely taken in account when considering the outcome of musculoskeletal rehabilitation.

Antonovsky presented with his salutogenic theoretical model an explanation for the maintenance or improvement of an individual’s location on a health ease/dis-ease continuum [11]. The person’s “sense of coherence” (SOC) is the model’s core construct and consists of three components: comprehensibility, manageability and meaningfulness. It is defined as: ”a global orientation that expresses the extent to which one has a pervasive, enduring though dynamic feeling of confidence that 1) the stimuli deriving from one’s internal and external environments in the course of living are structured, predictable, and explicable; 2) the resources are available to meet the demands posed by these stimuli; and 3) these demands are challenges worthy of investment and involvement” [11].

This definition summarizes a whole series of protective resources in the sense of a generalized, enduring perception of the environment and one’s own life (with its cognitive and affective components) and a dynamic optimism in the face of failure [12]. According to Antonovsky, the strength of the SOC has direct physiological consequences, affects health status and is associated with physical and psychological health. A strong SOC should be associated with a health promoting behavior, have an impact on the endocrine and immunological systems and should therefore mobilize the appropriate bodily resources [13].

Osteoarthritis was associated with a low SOC score in a group of old people aged 85 – 103 years [14]. In individuals with at least one diagnosed rheumatic disease, a strong SOC predicted a better outcome in the SF-36 subscales [15]. A high score on the SOC subscale “meaningfulness” was related to a better therapeutic outcome and better response to treatment in terms of pain intensity in chronic pain patients [16]. In the acute setting, SOC was positively associated with independence prior to admission [17] and with functional status at admission [18] but not with functional recovery.

Therefore, the SOC may serve as a predictor for rehabilitation outcome of patients with hip and knee OA. It may be able to specify individuals who will benefit more from treatments than others. Furthermore, treatment strategies and rehabilitation program content could be optimized based on more detailed knowledge about the influence of the SOC on health state and rehabilitation effects in osteoarthritis of the hip and knee.

To the best of our knowledge, there are no studies that analyzed the relationship between the SOC and the effect of a rehabilitation program on patients with hip or knee OA. The objective of this study was to investigate the relationship between the SOC and the course of physical and mental outcomes for patients with hip and knee OA before and after a comprehensive, in- or outpatient rehabilitation program. According to the concept of Antonovsky, our hypothesis was that a strong SOC would independently correlate with health status before rehabilitation and would predict short term improvement of physical and psychosocial functions after rehabilitation.

## Methods

### Patients

Patients with hip or knee OA were consecutively referred to the rehabilitation clinic “RehaClinic”, Bad Zurzach, Switzerland for in- or outpatient rehabilitation by their family physician or rheumatologist. They were invited to participate in the study if they fulfilled the criteria of the American College of Rheumatology for hip or knee OA [19,20]. Patients were excluded if they 1) had a history of medication abuse or non-compliance to therapies in the in- or outpatient program, 2) had insufficient German language skills to complete the assessment tools, 3) suffered from a severe illness (such as terminal cancer, chronic heart failure, severe asthma, moderate or severe dementia that prevented them from continuing therapy), or 4) did not want to participate in the study. During follow-up patients were excluded if they 1) underwent joint arthroplasty, 2) had a severe illness (as mentioned in point 3 above), 3) died, 4) refused further participation, 5) did not return the questionnaires or 6) if their questionnaires were incomplete according to the “missing rules” of the SF-36 or WOMAC [5]. The study’s protocol was approved by the Local Research Ethics Committee of the Health Department in Aarau, canton Aargau, Switzerland (EK AG 2008/026) and written informed consent was obtained from all participants.

### Intervention

Prior to inpatient rehabilitation of this study, outpatient physiotherapy was done in an institution of the patient’s choice. Reasons for inpatient management were reduced mobility due to severe osteoarthritis and high number of comorbidities that prevent from travelling frequently to outpatient therapies. The Swiss health insurance companies granted payment for inpatient rehabilitation only to those who were still suffering from osteoarthritis symptoms after completion of four series of outpatient physiotherapy with 9 treatments each and still requiring further treatment. The comprehensive multidisciplinary inpatient rehabilitation lasted 3–4 weeks. During rehabilitation NSAID and analgesics were minimized as far as possible. A more detailed description of the inpatient program is given in [5].

Some participants of the outpatient therapy program were admitted without having received other therapies prior to the program. Other patients may have received therapies in an institution of their choice prior to the outpatient program at the clinic. The outpatient program lasted up to 3 months and was conducted by the same group of therapist as the inpatient program.

Inpatients as well as outpatients received comprehensive multidisciplinary rehabilitation. Rehabilitation included active and passive therapies, each lasting 30 minutes. Rehabilitation was somatically oriented, did not include psychological interventions and was not based on a possible influence of the SOC on the effects of the rehabilitation.

Active therapies comprehend somatic exercise intervention provided by physiotherapists for improving physical capacities and functional performances. The standard active components of both programs were individual physiotherapy (e.g. movement against manual resistance, assisted range of motion exercise) and physiotherapy in groups (e.g. strengthening and endurance training, hydrotherapy, swimming with flippers).

Passive therapies included thermal therapy (fango packs), patient education (e.g. information about OA, coping with pain and disability), massage (optional) and electrotherapy (optional). Education was rather information than cognitive behavioral therapy and was therefore, classified as passive therapy. In each individual therapy session the patient’s general health condition was taken into account for the choice and intensity of the therapeutic intervention. Each patient was trained to continue an individual home rehabilitation program after discharge.

### Measures

Potentially confounding socio-demographic parameters, such as sex, age, and formal education were recorded on a standardized form [21]. The comorbidity (excluding joint disease) was entered on the Self-administered Comorbidity Questionnaire (SCQ) and by review of the medical records [22]. The exact number of active and passive therapies was individually calculated based on the schedule of each patient’s therapies.

The SOC was measured with the global score of Antonovsky’s short 13-item scale, the original SOC-13 questionnaire [23]. This is an abbreviated version of the original with 29 items reduced to 13 items [24,25] with good reliability and validity [26]. The questionnaire reflects a person’s capacity to cope in a stressful situation and addresses the perception of the environments’ comprehensibility (4 items), manageability (5 items) and meaningfulness (4 items) [24]. The questions are rated on a 7 point scale (1 = worst, 7 = best) with item-specific extremes, such as “never had this feeling” to “always had this feeling” or “completely fascinating” to “deadly boring”. Items 1, 2, 3, 7 and 10 are formulated negatively (1 = best, 7 = worst) and have to be reversed for scoring. The total score ranges from 13 (weakest) to 91 (strongest SOC). The total score is the unweighted mean of at least 9 of the 13 (= 69%) completed items and was transformed to a scale of 0 (= no SOC) to 100 (= best possible SOC). This “2/3 rule“ was arbitrarily chosen analogue to other scoring calculations for outcome instruments because no missing rule was reported in the literature for the original questionnaire [27]. The SOC questionnaire has been used in at least 32 countries and 33 languages and in studies covering populations of 20000 persons. Being used all over the world, the SOC questionnaire seems to be a cross culturally applicable instrument [26,28].

The Western Ontario and McMaster Universities Osteoarthritis Index (WOMAC) is a disease-specific multidimensional self-assessment instrument for OA of the lower extremity. We used the validated German version [29]. It measures pain (5 items), stiffness (2 items) and physical functional ability (17 items) leg- but not joint-specifically [30]. All 24 items were evaluated on a numeric rating scale ranging from 0 (“no symptoms/no limitation’’) to 10 (“maximal symptoms/maximal limitation’’). According to the missing rule of the user’s guide, the subscores can only be determined if at least 4 of the 5 pain items, 1 of the 2 stiffness items, and 14 of the 17 function items are completed fully [30].

The Short Form 36 (SF-36) comprehensively measures the dimensions of quality of life, physical, mental and psychosocial health [31]. We used the validated German version [32]. The psychometric quality of the SF-36 has been proven in various settings, also in patients with rheumatic conditions [33]. The questionnaire contains 36 items in 8 scales (see Table 1). According to the SF-36 manual, the three best scales that yield the most valid measures for the mental component of health status are mental health, role emotional and social functioning [31]. The SF-36 scales vitality and general health have moderate empirical validity for both physical and mental health outcomes. Physical functioning, role physical and bodily pain have substantial validity as measures of physical health status.

**Table 1:** Baseline, outcome and correlation data of SOC-13, SF-36 and WOMAC

		**Baseline**		**Discharge**		**Effect (Baseline** → **Discharge)**			**Correlation with SOC**		
		**m**	**s**	**m**	**s**	**m**	**s**	**ES**	**r Baseline**	**r Effect**	**FSC MCS**[31]
**SF-36**	Physical functioning	36.2	20.2	40.4	20.9	4.2	17.4	0.21	0.10	0.00	−0.22999
	Role physical	20.6	30.6	25.7	35.9	5.1	32.0	0.17	0.17	−0.02	−0.12329
	Bodily pain	26.7	16.8	35.7	18.1	9.0	16.7	0.53	0.14	−0.01	−0.09731
	General health	53.7	18.8	56.1	18.7	2.5	14.0	0.13	0.34	−0.07	−0.01571
	Vitality	41.6	20.0	47.2	19.2	5.6	16.5	0.28	0.32	−0.04	0.23534
	Social functioning	64.1	27.5	69.1	25.7	5.0	21.1	0.18	0.42	−0.19	0.26876
	Role emotional	52.3	45.0	54.3	46.2	2.0	46.4	0.05	0.44	−0.14	0.43407
	Mental health	61.9	20.1	67.0	18.3	4.9	14.2	0.25	0.53	−0.11	0.48581
	PCS	31.7	7.6	33.8	7.8	2.1	6.3	0.28	−0.08	0.03	
	MCS	46.7	12.8	48.5	11.7	1.8	9.6	0.14	0.57	−0.18	
**WOMAC**	Pain	51.8	21.3	63.1	22.5	11.3	17.9	0.53	0.25	0.00	
	Stiffness	50.0	25.2	58.7	24.1	8.8	23.0	0.35	0.18	−0.04	
	Function	51.7	21.3	60.7	21.6	9.0	15.6	0.42	0.23	−0.03	
	Global	51.6	20.5	61.0	21.1	9.4	15.3	0.46	0.24	−0.03	
**SOC-13**	Global Score	69.8	16.3								

Legend: *m* = arithmetic mean, *s* = standard deviation, *ES* = effect size, *r* = correlation, *SF-36* = short form 36, *FSC* = Factor Score Coefficients, *PCS* = physical component summary, *MCS* = mental component summary, *WOMAC* = Western Ontario and McMaster Universities Index, *SOC-13* = Sense of coherence, 13-item version.

This order of weight regarding mental health is reflected by the level of the factor score coefficients (FSC), which are the weights for the linear composition that determines the SF-36 Mental Component Summary (MCS) [31]. The higher the FSC the more “mental” the SF-36 score is (see Table 1, last column). Vice versa, the level of the FSC of the SF-36 Physical Component Summary (PCS) reflects how “physical” a SF-36 score is [31].

### Analyses

WOMAC and SF-36 were measured in the clinic on entry to (baseline) and on discharge (follow-up) from inhouse rehabilitation and accordingly at the beginning (baseline) and after completion (follow-up) of the outpatient program. The SOC-13 questionnaire was assessed at baseline. All analyses were performed using the statistical software package SPSS 20.0 for Windows (SPSS Inc., Chicago, IL, USA).

All instrument scores were transformed into a scale ranging from 0 (“maximal symptoms/maximal limitation”) to 100 (“no symptoms/no limitation”) to facilitate comparison of the descriptive data [27]. Standardized effect sizes were calculated to obtain a quantification of the score changes or “effects” of the intervention. Effect sizes (ES) of 0.00– 0.19 reflect very small, 0.20-0.49 small, 0.50-0.79 moderate, and 0.80 or more large improvement [34]. Negative ES mean a worsening of health.

Baseline scores and the observed effect sizes after rehabilitation of the SF-36 scales and WOMAC were correlated with the baseline SOC-13 with bivariate non-parametric rank correlation coefficient according to Spearman. These results express an association that is not controlled for possible confounders. These correlations of the SF-36 scales were compared to the FSC for the SF-36 MCS, which quantifies the factor load on the psychosocial dimension.

These associations were further examined with stepwise multivariate linear regression considering the effects of potential confounders. The baseline score of the SF-36 and WOMAC scales and accordingly the score change (baseline → discharge) was defined as the dependent variable. Included as the independent variables for the baseline score were the joint (hip/knee), type of treatment (inpatient/outpatient), sex (m, f), age (years), living with partner, sports, number of comorbidities, and the SOC-13 global score. For the calculation of the effect (baseline → discharge), the corresponding score at baseline, being the most important predictor for a change [35], was included as independent variable as well as the total number of active and passive therapies, the length of treatment, and all other potential confounders as listed above.

## Results

### Patients

A total of 350 patients were referred consecutively to the rehabilitation clinic “RehaClinic” in Bad Zurzach, Switzerland and recruited to the study. 8 patients had to be excluded for reasons of non-compliance. A further 3 patients were excluded because of incomplete data according to the “missing rules” above. In total, complete data was available for 335 patients, 136 (41%) with hip and 199 (59%) with knee OA.

Socio-demographic variables, disease-relevant characteristics at baseline and details about the in- and outpatient program are given in Tables 2 and 3. Overall, outpatient rehabilitation lasted longer but included a smaller number of hours of active therapies. The inpatient program contained more individual active aquatic therapies and more active land-based therapies in groups. Passive group therapies in the water were not provided. The total duration of therapy in days includes days with no compulsive therapies in the inpatient rehabilitation. Inpatients were instructed to non-supervised strength and endurance training between planned therapies and motivated to attend aquatic exercise groups on weekends.

**Table 2:** Overall socio-demographic and disease-relevant characteristics at baseline

		
Female (%)		68.9
Hip (%)		41.4
Knee (%)		58.6
Inpatient (%)		74.0
Outpatient (%)		26.0
Education (%)	Basic school (8–9 years)	34.6
	Vocational training	48.0
	College/high school/university	16.9
Partnership (%)	lives alone	31.7
	lives with partner	67.7
Comorbidities (%)	none	0.3
	1	8.0
	2	21.6
	3	27.9
	4 and more	42.2
Sports (%)	none	51.1
	≤1 hours/week	15.7
	1-2 hours/week	18.0
	≥2 hours/week	14.3

Legend: All percentage data are from the “complete data at follow-up” groups.

**Table 3:** Joint and type of treatment-specific characteristics at baseline

	**Inpatient**		**Outpatient**	
	**Hip**	**Knee**	**Hip**	**Knee**
**Patient numbers (n):**				
Included at baseline	92	167	53	38
Dropped out/incomplete data	6	3	2	0
Complete data at follow-up	86	164	51	38
**Mean (standard deviation):**				
Age (years)	64.0 (10.7)	65.2 (10.5)	64.3 (9.8)	67.8 (9.1)
Duration of therapy (days)	22.0 (4.1)	22.3 (3.6)	36.4 (28.3)	31.2 (28.0)
**Mean hours of therapies, totally (standard deviation):**				
Active, totally	23.6 (9.2)	23.4 (9.0)	10.3 (6.7)	10.7 (5.6)
Active, individual, land-based	5.2 (2.0)	5.2 (1.8)	4.8 (2.6)	4.5 (2.0)
Active, individual, aquatic	6.8 (5.4)	7.1 (5.3)	1.3 (3.1)	1.8 (3.9)
Active, in groups, land-based	8.7 (4.4)	8.7 (4.6)	2.2 (2.1)	2.4 (2.5)
Active, in groups, aquatic	2.8 (1.7)	2.4 (1.8)	2.0 (2.3)	2.1 (1.8)
Passive, totally	5.6 (4.2)	6.2 (5.7)	5.1 (4.3)	5.0 (5.8)
Passive, individual, land-based	4.1 (3.9)	4.5 (5.4)	4.8 (4.2)	4.9 (5.8)
Passive, individual, aquatic	0.5 (1.3)	0.5 (1.3)	0.2 (0.8)	0.1 (0.3)
Passive, group, land-based	1.0 (1.3)	1.2 (1.6)	0.1 (0.7)	0.0 (0.2)

Legend: All mean (standard deviation) data are from the “complete data at follow-up” groups.

### Score changes

The instrument scores and changes after rehabilitation are shown in Table 1. All health dimensions show improvements from entry to discharge with ES from 0.05 to 0.53 (pain). The improvement in pain was moderate (ES = 0.53) on both SF-36 and WOMAC. Small improvements were reflected by SF-36 physical functioning, vitality, mental health, and MCS as well as by stiffness, function, and global on the WOMAC.

### Correlation and regression analysis

The Spearman correlation coefficient of the SOC-13 global score with the baseline scores of SF-36 and WOMAC showed correlations ranging from lowest at 0.10 on physical functioning, increasing to 0.32 on vitality, and highest at 0.53 on mental health (Table 1). The correlations of the effects (baseline → discharge) on the SF-36 scales with the SOC-13 global score were all negative and varied between 0.00 (physical functioning) and −0.19 (social functioning). This means that patients with a strong SOC (and, by that, a high baseline level of social functioning) tended to have lower improvement in social functioning.

The results of the multivariate regression models are reported in Table 4. The whole regression model explained between 12.9% (role physical) and 29.8% (mental health) of the total variance at baseline. At follow-up, the corresponding numbers for the SF-36 effects were 13.5% and 28.3%. These gradients were consistent when looking at the variance explained by the baseline SOC-13.

**Table 4:** Explained variance of baseline values and effects of SF-36 and WOMAC by SOC-13 global score in a multivariate linear regression model

		**Baseline**		**Effect (Baseline → Discharge)**	
		**Explained Variance by SOC-13 (%)**	**Total Variance (%)**	**Explained Variance by SOC-13 (%)**	**Total Variance (%)**
**SF-36**	Physical functioning	< 0.1	16.4	0.1	15.4
	Role physical	1.4	12.9	< 0.1	13.5
	Bodily pain	0.2	13.7	0.3	18.3
	General health	12.2	20.5	0.1	15.5
	Vitality	11.8	20.3	2.1	26.5
	Social functioning	16.8	19.5	< 0.1	25.5
	Role emotional	19.8	21.4	0.7	26.9
	Mental health	27.9	29.8	4.6	28.3
	PCS	2.5	19.3	< 0.1	15.1
	MCS	32.8	34.4	2.1	26.7
**WOMAC**	Pain	4.4	13.9	0.7	14.0
	Stiffness	1.0	11.5	0.7	25.6
	Function	2.0	16.4	0.6	12.2
	Global	2.4	17.2	0.5	10.8

Legend: *SF-36* = short form 36, *PCS* = physical component summary, *MCS* = mental component summary, *WOMAC* = Western Ontario and McMaster Universities Index, *SOC-13* = Sense of coherence, 13-item version.

Confounders included in 3 or more multivariate regression models at baseline were sex (m, f), number of comorbidities, sports and the type of treatment (inpatient/outpatient). These confounders explained between 0.7% and 2.2% of the total variance for sex, 1.4% and 4.6% for the number of comorbidities, 0.6% and 2.7% for sports and 1.5% and 10.5% for the type of treatment, respectively. For the calculation of the effects, the corresponding score at baseline (explained variance between 23.0% and 54.0%) and the total number of passive therapies (0.5% and 1.2%), type of treatment (0.5% and 1.4%), age (0.5% and 1.5%) and the number of passive therapies (0.5% and 1.2%) were included in 3 or more models. All other potential confounders as listed above were stepwise excluded to obtain the final regression model when the explained change was not significant (less than 0.6% to 1.7% of the maximum of variance at baseline and 0.5 to 1.4% for the effect models).

At baseline, 93.6% (27.9%/29.8%) of the total variance of baseline SF-36 mental health was explained by the baseline SOC-13. On the other hand, the SOC score at baseline did not contribute to the explanation of the total variance on the physical dimensions (baseline scores).

Looking at the FSC of the MCS reflecting the “mental” load of each scale (Table 1), there is a quasi perfect positive linear relationship to the correlations between the SOC-13 and the SF-36 baseline scores by a Spearman correlation coefficient of 0.95 (1.00 = perfect). There is further a negative linear relationship between the FSC and the correlation coefficients of the SOC-13 to the effects of the SF-36 scales by the high Spearman correlation of −0.86. Looking at the gradients of explained variance described in Table 4, the FSC correlates to the total variance by 0.83 at baseline and 0.93 to the effects at the follow-up. The corresponding correlations of the FSC to the variance explained by the baseline SOC-13 were 0.95 at baseline and 0.61 at follow-up.

## Discussion

This prospective cohort study analyzed the correlation of Antonovsky’s SOC to physical and mental health dimensions in patients with hip or knee OA before and after in- and outpatient rehabilitation. The hypothesis that a strong SOC independently correlates to mental health could be confirmed. There was an almost perfect dose-relationship between the “mental” load of the SF-36 scales and the baseline SOC-13 with correlations up to 0.95. The hypothesis that a strong SOC also independently correlates to physical health, as stated by Antonovsky, could not be confirmed.

According to Antonovsky’s salutogenic concept, a strong SOC is associated with physical and psychological health [13]. A strong SOC should be associated with health promoting behavior and should mobilize the appropriate bodily resources. Therefore, perceived health should become better which will positively influence the physical dimensions of health-related quality of life [26]. This can be measured by the SF-36 and the WOMAC, whereas health promoting behavior cannot be directly measured by these instruments.

Our results showed relatively high correlations of the SOC with the psychosocial and psychological health dimensions at baseline which corresponds to the concept of Antonovsky. In contrast to that, the low correlations of the SOC with the level of physical health at baseline did not reflect the hypothesis of Antonovsky. A stronger SOC seems to have a stronger influence on mental than on physical health at baseline and less, but also when looking at the effects after rehabilitation. This is summarized by the high correlation of the SOC-13 to the SF-36 MCS (0.57) compared to the SF-36 PCS (−0.08) at baseline.

Our findings are in line with studies as summarized in the reviews by Flensborg-Madsen et al. [36,37]. High correlations were found for the SOC and health measures that incorporate psychological and psychosocial aspects (0.46 to 0.76), stress and behaviours whereas contradictory results were found in physical health (−0.46 to +0.51). Another study with patients affected by non-specific musculoskeletal disorders reported that the SOC was correlated to all mental dimensions of the SF-36 (social functioning, role emotional and mental health) but not to the physical dimensions (physical functioning, role physical and bodily pain) [38]. The SOC was inversely correlated with Arthritis Impact Measurement Scales (AIMS) anxiety and depression scores in patients with rheumatic disorders [39] and a weak SOC seems to be associated with increased prevalence of depression [40]. Few significant differences in physical status between subgroups with a stronger versus a weaker SOC were found in hip fracture patients [17]. Those with a weaker SOC stayed longer in the hospital and had to rely more on assistance before admission to the hospital. Furthermore, several studies that show no correlation or only weak correlations might not have been published for that reason (publication bias) as hypothesized in [36].

A systematic review found contradictory results in longitudinal studies examining the predictive value of the SOC for treatment outcome [28]. The SOC was able to predict outcome in some studies, not in others. In a Swedish rheumatology clinic, a strong SOC was one of the most important factors predicting better outcome after rehabilitation in Health-related Quality of Life (HRQL) in people with rheumatic diseases in several SF-36 subscales [15]. After an inpatient rehabilitation program focusing on physical training, medical help and individual support and advice for self-care, a positive outcome in seven of the eight SF-36 subscales was predicted by a strong SOC [15]. In the acute hospital, functional status at admission was significantly associated with the SOC whereas functional recovery was only significantly associated with motivation, but not with the SOC [18]. In patients with non-specific musculoskeletal disorders, the SOC was not a strong predictive instrument for treatment outcome after 4–5 months of treatment with Body Awareness Therapy or Feldenkrais [38]. These differences may result from the different focus of treatments.

The high correlation of the SOC to SF-36 mental health (r = 0.53) and MSC (r = 0.57) raises the question of a construct overlap of the psychosocial components of SF-36 and the SOC-13. To the best of our knowledge, there is no study that specifically examined the construct overlap or differences of the SOC to the mental health measurement dimensions of SF-36. Our data suggest that these two constructs are strongly related. This is in accordance with the conclusion that mental health and mental wellbeing are close to the SOC, and the construct of mental health is a correlated, but independent construct [26].

According to Antonovsky, a person’s SOC develops during adolescence and remains stable during adulthood [25]. Following this concept, we only measured the SOC at baseline of this study and assumed that it remained stable during rehabilitation. However, more recent research suggests that the SOC tends to increase with age through the whole life span and does not seem to be as stable as Antonovsky assumed [28]. Over a period of five years, the SOC was only stable for persons with initially high levels of SOC. For persons with a weaker SOC, individual conditions and societal changes influenced their SOC [41]. Even in the short term of acute care rehabilitation, the SOC changes after multiple trauma [42].

Prediction in life increases with age because life experience increases and uncertainty of life time ahead decreases with age. By that, the SOC increases with age because predictability is part of SOC’s comprehensibility [37]. This finding could be a possible explanation for the difference in the association with physical and psychosocial health. With greater age, the SOC increases although older persons have increasing somatic health disorders [28]. In this sense, age is an important confounder for the SOC. In our setting, age correlated with the SOC, even after controlled for above described confounders (data not shown in detail). However, one study of a large German population showed a slight decrease of the SOC with increasing age [23].

The average SOC score in our setting was similar to that of other OA samples and to samples with various other disorders [14]. The affected mobility by OA and the consequences of limitation in physical and socio-cultural activities may contribute to a lower SOC score compared to age-related groups. Our study replicated this finding by an association of a low SOC score to weak social functioning. These subjects experienced, on average, higher improvements in social functioning after rehabilitation (negative correlation to the SOC score).

In this study, in a homogeneous setting of inpatient and outpatient as well as knee and hip treatment at the same clinic, the same group of therapists treated all patients with a very similar, homogeneous therapy program for all patients. This is a strength of the study because the outcome effects are supposed to depend less on the variability of treatment. A further strength is the large number of knee and hip OA patients participating in this study. A limitation of the study is that outpatients and hip OA were under-represented in the study due to the observational design. The SOC was only assessed at baseline.

## Conclusion

The SOC showed a strong association with psychosocial health but a weak association with physical health. In the SF-36 scales, the dependency of a scale on the SOC was highly associated with the amount of psychosocial characteristic/load of the scale. This relationship was stronger for baseline health than for health change after rehabilitation. Antonovsky’s concept that a strong SOC should positively influence psychosocial health could be confirmed but not that it does the same to physical health.

## Abbreviations

AIMS: Arthritis impact measurement scale; ES: Effect size; EULAR: European league against rheumatism; FSC: Factor score coefficients; HRQL: Health-related quality of life; MCS: Mental component summary; OA: Osteoarthritis; PCS: Physical component summary; SCQ: Self-administered comorbidity questionnaire; SF-36: Short form 36; SOC: Sense of coherence; SOC-13: Sense of coherence 13 items questionnaire; WOMAC: Western Ontario and McMaster Universities Osteoarthritis index.

## Competing interests

The authors declare that they have no competing interests.

## Authors’ contributions

All authors commented on the draft, read and approved the final manuscript. TB wrote the original manuscript, helped in the data analysis, and contributed to the interpretation and presentation of the data. FA was responsible for the analysis and the interpretation of the data and contributed to all parts of the work of this study. SL was responsible for the data acquisition, helped in the analysis and the interpretation of the data. AA was responsible for the conception, the design, and the resources for the study and helped to prepare the manuscript. All authors read and approved the final manuscript.

## Pre-publication history

The pre-publication history for this paper can be accessed here:

http://www.biomedcentral.com/1471-2474/14/159/prepub
